# Using noninvasive ventilation to prevent extubation failure: it is good news, but do we really know what “high risk” means?

**DOI:** 10.1186/s13054-016-1336-z

**Published:** 2016-07-04

**Authors:** Alastair J. Glossop, Antonio M. Esquinas

**Affiliations:** Department of Critical Care, Sheffield Teaching Hospitals NHS Foundation Trust, Herries Road, Sheffield, S5 7AU UK; Intensive Care and Non Invasive Ventilatory Unit, Hospital Morales Meseguer, Avenida Marques de Los Velez, Murcia, 30.008 Spain

We read with great interest the article by Thille et al. [1] and commend the authors on their work. It is accepted that noninvasive ventilation (NIV) used prophylactically reduces post-extubation respiratory failure, but its impact on reintubation remains contentious [2]. Therefore, the current study, which reports reduced rates of reintubation from 28 % to 15 % in high-risk populations with NIV use, is welcomed.

The study has several limiting factors. It is a prospective before–after study across a long time period, and the authors acknowledge this potential source of bias. There is a large gap between patient cohorts, with data from the original cohort collected between 2005 and 2006 and data from the “after” group collected between 2010 and 2012. No explanation is provided as to why this second period—4 years later—was chosen, which raises concerns regarding selection bias. Additionally, data were collected from a single centre with extensive experience of NIV use, an important factor in the success of NIV use post-extubation [3].

In mitigation, no difference between baseline reintubation rates of low-risk patients was seen between the two groups, and the second patient cohort contained fewer surgical patients—a group known to benefit from post-extubation NIV use [4].

We applaud the definition of extubation failure as reintubation within 7 days rather than 48–72 h, as we feel this provides a pragmatic view. It is likely to result in greater numbers of extubation failures and makes the findings more impressive. We also feel that an average time of NIV use of 8 h within the first 24 h represents a reproducible period for prophylactic use in critical care patients, in whom treatment compliance may be an issue.

We feel that this is an important study and welcome its contribution to the evidence pool. It does, however, raise the important question of what constitutes “high risk” of extubation failure. The authors state that NIV is “pointless” as a prophylactic measure in low-risk populations, but define over 60 % of patients included as high risk; as demands on frontline services increase this figure is surely set to rise. The emergence of high-flow nasal cannula (HFNC) therapy in patients defined as low risk of extubation failure [5] brings an even sharper focus on the need for risk stratification in all ICU patients, and further work is warranted to help guide post-extubation management.

## Abbreviations

HFNC, high-flow nasal cannula; ICU, intensive care unit; NIV, noninvasive ventilation.
